# Possibility of Human Gender Recognition Using Raman Spectra of Teeth

**DOI:** 10.3390/molecules26133983

**Published:** 2021-06-29

**Authors:** Ozren Gamulin, Marko Škrabić, Kristina Serec, Matej Par, Marija Baković, Maria Krajačić, Sanja Dolanski Babić, Nikola Šegedin, Aziz Osmani, Marin Vodanović

**Affiliations:** 1 Department of Physics and Biophysics, School of Medicine, University of Zagreb, 10000 Zagreb, Croatia; ozren@mef.hr (O.G.); marko.skrabic@mef.hr (M.Š.); maria.krajacic@mef.hr (M.K.); sanja.dolanski.babic@mef.hr (S.D.B.); nikola.segedin@mef.hr (N.Š.); 2 Center of Excellence for Advanced Materials and Sensing Devices, Research Unit New Functional Materials, 10000 Zagreb, Croatia; 3 Center of Excellence in Reproductive and Regenerative Medicine, School of Medicine, University of Zagreb, 10000 Zagreb, Croatia; 4 Department of Endodontics and Restorative Dentistry, School of Dental Medicine, University of Zagreb, 10000 Zagreb, Croatia; mpar@sfzg.hr; 5 Institute of Forensic Medicine and Criminalistics, School of Medicine, University of Zagreb, 10000 Zagreb, Croatia; mbakovic@mef.hr; 6 Community Health Center “Kutina”, 44320 Kutina, Croatia; osmani.aziz@hotmail.com; 7 Department of Dental Anthropology, School of Dental Medicine, University of Zagreb, University Hospital Centre, 10000 Zagreb, Croatia; vodanovic@sfzg.hr

**Keywords:** Raman spectroscopy, multivariate statistical methods, principal component analysis, support vector machine, artificial neural network, forensic dentistry, gender determination

## Abstract

Gender determination of the human remains can be very challenging, especially in the case of incomplete ones. Herein, we report a proof-of-concept experiment where the possibility of gender recognition using Raman spectroscopy of teeth is investigated. Raman spectra were recorded from male and female molars and premolars on two distinct sites, tooth apex and anatomical neck. Recorded spectra were sorted into suitable datasets and initially analyzed with principal component analysis, which showed a distinction between spectra of male and female teeth. Then, reduced datasets with scores of the first 20 principal components were formed and two classification algorithms, support vector machine and artificial neural networks, were applied to form classification models for gender recognition. The obtained results showed that gender recognition with Raman spectra of teeth is possible but strongly depends both on the tooth type and spectrum recording site. The difference in classification accuracy between different tooth types and recording sites are discussed in terms of the molecular structure difference caused by the influence of masticatory loading or gender-dependent life events.

## 1. Introduction

Identification, the process of establishing the identity of human remains, is the first step in any medicolegal investigation regarding the remains of unknown identity [1]. The need for identification of human remains arises in different settings of armed conflicts, natural mass disasters, and migrant crises as well as in everyday work of forensic doctors. The importance of identification of human remains has ethical and humanitarian aspects as well as administrative. In forensic cases, without establishing the identity, no meaningful investigation can be conducted.

The identification process differs wildly with respect to the state of the remains. In cases of skeletal remains, the first step is establishing the biological profile—Defining physical characteristics of individual: ancestry, gender, age, and stature. Because many methods of age and stature recognition are gender dependent, gender recognition is the essential and primary part of any identification [2]. Establishing the gender of the remains is not only necessary in skeletal remains, but also when only body parts are available for analysis.

Gender recognition is based on sexual dimorphism of skeleton between male and female, pertaining mainly to the size and shape of the body as well as to the differences in growth and development pattern. So far, three different groups of gender recognition techniques are available—morphological, osteometric, and biological. Morphological methods use visual examination of the presence or degree of expression of qualitative traits that are more or less specific for female or male sex. The pelvis and skull are the bones mostly used in this group of methods with reliability of over 95% in complete pelvis and approximately 80% with skull only [3]. These methods tend to be subjective, are prone to decrease in accuracy in incomplete or fragmented remains and are highly dependable on the investigators experience. Osteometric methods use quantitative measures, usually breadth and length of bones and parts of bones, which are significantly different within male and female sex. Osteometric methods are more objective in essence, but often suffer from intra and inter observer discrepancies [4]. Biological (molecular) methods use polymerse chain reaction (PCR) techniques of DNA amplification and most often analyze the amelogenin gene, the SRY gene on the Y chromosome, and the DYS14 gene [5]. These are the most reliable gender recognition methods, but also most expensive and time consuming.

The importance of gender recognition as well as the difficulties encountered on the way are best illustrated by the extent of research conducted on the topic. Almost every bone was the subject of either morphologic or osteometric assessment and, at this point, there are no field-wide best practices and guidelines that indicate which method should or should not be used to estimate gender [6]. Since a great amount of forensic or archeological skeletal material is either incomplete, fragmentary, damaged, commingled, burned, immersed in sea, and so on, various methods are helpful at some point. Subsequently, the fact is that we often use several different methods in order to estimate gender as accurately as possible in given circumstances. In addition to the above-mentioned disadvantages, sexual dimorphism of skeletal remains of subadults (until the end of puberty) is far less pronounced and gender recognition represents at the very least a challenge, and often is simply inadequate without using biological methods [7].

A great number of research analyzed teeth in order to investigate the possibility of gaining information useful for the identification process. In incomplete, fragmentary, damaged, commingled, or burned remains, very often teeth are present and preserved. Teeth are the hardest, most lasting tissue of the human body and are usually found undamaged [8]. In addition, in forensic and archeological scenarios often only the skull is available for the analysis. The conducted research established that numerous useful information could arise from teeth analysis—gender and age recognition, ancestry, and even stature prediction [9]. As stated by several studies, teeth show a high degree of sexual dimorphism, and different morphological and odontometric methods of gender recognition are in use [10].

Forensic dentistry employs a wide range of analytical tools for gender determination [11,12,13,14,15]. The traditional methods are based on quantitative or qualitative evaluations of craniofacial and dental morphological features [16]. Dental morphometric gender-determination methods commonly include regression analyses of mesiodistal and buccolingual tooth dimensions, or identification of the presence of certain gender-specific morphological features on crowns of canines or mandibular molars [10]. Unlike craniofacial differences that are markedly pronounced between genders, sexual dimorphism in teeth is less distinctive, leading to lower reliability of forensic dental gender-determination methods. Generally, the reliability of tooth morphometric methods is population-dependent and impaired by high within-group variability, which limits their accuracy [17]. More accurate gender determination methods based on tooth samples include the identification of the Barr body from dental pulp [18], amplification of X- or Y-chromosome specific DNA sequences using polymerase chain reaction [19], or identification of gender-specific differences in enamel matrix protein, amelogenin [20]. Despite being virtually 100% accurate, these molecular analysis methods require destructive specimen preparation and irreversible consumption of tooth tissues. A more conservative, non-destructive analysis such as that offered by Raman spectrometry performed on outer tooth surfaces without any previous sample preparation has clear advantages for forensic purposes.

Results of our previous work [21] regarding age determination by using Raman spectra of teeth indicated the existence of differences between spectra of females and males. In the present work, this fact was utilized by building gender recognition models i.e., for distinguishing male and female teeth. For that purpose, Raman spectra from different tooth types recorded at two different sites (apex and anatomical neck) were used for classification model generation with two different algorithms, support vector machine (SVM) and artificial neuron network (ANN).

## 2. Materials and Methods

### 2.1. Teeth Sampling

The teeth belong to the collection of the Department of Dental Anthropology School of Dental Medicine University of Zagreb. As an integral part of the regular practice of informed consent at the clinic, all donors agreed that their teeth can be retained and used for research. No personal data and other identifying information about donors have been disclosed to the investigators. The collection and handling of biological material were conducted in full accordance with the World Medical Association Declaration of Helsinki regarding ethical principles for medical research involving human subjects. The soft tissues that remained after tooth extraction were removed using a plastic brush and the teeth were disinfected by soaking in a 1% formaldehyde solution for 24 h. Thereafter, the teeth were stored dry in dark containers at room temperature (23 ± 3 °C) in the archive of the Department of Dental Anthropology of the School of Dental Medicine, University of Zagreb, Croatia. The sample of 55 teeth (19 premolars and 36 molars) used for this study was obtained by a random draw from the aforementioned archive (Table A1, Appendix A). The age of tooth donors ranged between 11 and 76 years. The teeth had been extracted due to various indications, the most common being periodontitis and failed endodontic treatment. To simulate a forensic analysis of teeth at different post-extraction time periods, the time span between extraction and performing Raman spectroscopic measurements ranged between 0.1 and 5.5 years. No special selection criteria (either inclusion or exclusion) were applied; teeth affected with various pathological processes were deliberately included to simulate a realistic sample.

### 2.2. Raman Measurements and Pre-Processing

Raman spectra were recorded using an FT-Raman accessory of the Spectrum GX spectrometer (Perkin-Elmer, Waltham, MA, USA) equipped with an Nd:YAG laser with a wavelength of 1064 nm. Each spectrum was recorded by averaging 100 scans in the spectral range between 3500 and 200 cm^−1^ and with a spectral resolution of 4 cm^−1^. Spectra were collected from two distinct sites on each tooth: apex and distal part of anatomical neck. At each tooth site, an area of 2.5 mm in diameter was chosen, over which the excitation laser spot (0.25 mm in diameter) was moved in a scanning motion to collect Raman spectra from 10 different positions i.e., spots. This was done in order to account for local heterogeneities of mineralized tooth tissues. Therefore, a total of 20 spectra per tooth were collected (2 sites × 10 spectra). Finally, several spectra with low signal-to-noise ratio were removed. The spectra were stored in a dataset and connected with the donor’s gender and collection site. All spectra were baseline corrected and normalized using the peak at 960 cm^−1^ (symmetric PO_4_ stretching) to exclude possible differences caused by variations in recording conditions. Finally, to remove spectra variations mostly due to fluctuations of recording conditions, an advanced preprocessing technique, generalized least squares weighting (GLSW), was applied [8,22,23,24].

### 2.3. Statistical Analysis

In order to access the ability of gender recognition by Raman spectroscopy, multivariate statistical methods were used on Raman spectra of teeth; first, decomposition of original data by principal component analysis (PCA) (MATLAB R2010b, The Mathworks Inc., Natick, MA, USA, with its add-on PLS_Toolbox, Eigenvector Research, Manson, WA, USA), and second, classification of data by Support Vector Machine (SVM) and Artificial Neural Network (ANN) algorithms (RapidMiner Studio 9.9, RapidMiner GmbH, Dortmund, Germany). For data decomposition, pre-processed Raman spectra in the 3500–200 cm^−1^ region were used to build 6 distinct PCA models with 1–20 principal components; 4 models with respect to tooth type and collection site (molar apex, molar anatomical neck, premolar apex, and premolar anatomical neck) and 2 “joint” models with both tooth types mixed together (molar + premolar apex and molar + premolar anatomical neck). Data arrays utilized for PCA models included absorbance values of all the wavenumbers in the 3200–200 cm^−1^ region and the used parameters were the singular value decomposition (SVD) algorithm, cross validation venetian blinds with 10 splits, and the generalized least squares weighting (GLSW) filter declutter threshold set to 0.02 (program default setting). Next, the dimensionality reduction of spectroscopic data was obtained by selecting scores of first 20 principal components from each of the aforementioned PCA models. These first 20 PC scores were then used for further calculation i.e., classification of data with respect to donor’s gender by means of SVM and ANN algorithms. Four SVM and four ANN models with respect to tooth type and collection site were built: Molar apex, molar anatomical neck, premolar apex, and premolar anatomical neck (for each model 1–20 PCs was used). For the purpose of model assessment, spectra were divided into two datasets, where approximately 83% of all spectra were used as a calibration set, and the rest was used as a validation set. Additionally, all ANN and SVM models were cross-validated with the 10-fold cross-validation method. The parameter used for SVM was radial kernel type, parameter C was zero, and kernel gamma was 1, kernel cache was 200, convergence epsilon was 0.001, maximum number of iterations was 100,000, L_pos_ was 1, and L_neg_ was 1. ANN with back propagation was configured in three layers, number of nodes in the hidden layer was defined as 1 plus total number of attributes, and classes divided by 2. The activation function was sigmoid, learning rate was 0.3, momentum was 0.2.

Student’s *t*-test (STT) (software Kinetics, running under MATLAB R2010b) [25] was utilized to evaluate and emphasize the differences between male and female spectra recorded on molar apex. To obtain the difference spectrum, the mean spectrum of one sample group (male) was subtracted from the mean spectrum of another group (female). STT was used to analyze the difference spectrum in the manner that each wavenumber was examined to determine if the difference in intensity between mean spectra of two groups is statistically significant (*p* < 0.01). Moreover, STT was used to inspect a possible statistically significant difference (*p* < 0.05) between intensity ratios of three vibrational bands for two genders.

## 3. Results

**Figure 1:**
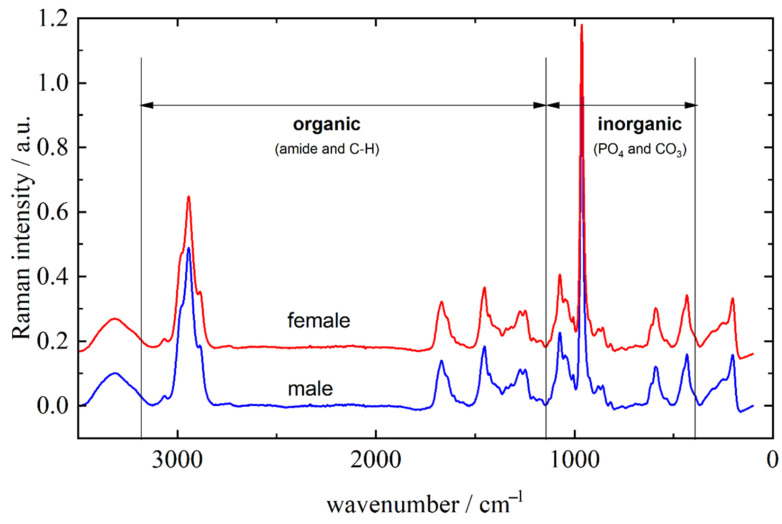
Representative Raman spectra of male and female molar teeth recorded from apex in the 3500–200 cm^−1^ range. The spectra are offset along the *y*-axis for clarity.

Representative Raman spectra of male and female molar teeth, recorded on apex, are presented in Figure 1. The inorganic part is approximately represented with vibrational bands between 400 and 1100 cm^−^^1^ (mostly PO_4_ and CO_3_ vibrations) while bands originating from the organic parts of the tooth can be found between 1100 and 3100 cm^−^^1^ (amide bands and C–H vibrations) [26,27]. It can be clearly seen from Figure 1 that male and female teeth cannot be distinguished solely on visual characterization of the Raman spectra. Furthermore, visual categorization of spectra with respect to the recording site is also virtually impossible as the tooth apex and anatomical neck have extremely similar spectral profiles (spectra not shown) for both male and female teeth.

Later in this article, after application of statistical methods, it will be shown that there exist small differences in inorganic/organic ratio of the several vibrational bands. These properties reflect the fact that the two observed types of tooth tissue are built from the same molecules but in different relative amounts. The major component of the inorganic part is carbonated calcium-deficient hydroxyapatite, while most of the organic part consists of collagen [28,29]. Assignation of the relevant vibrational bands is listed in Table 1.

**Figure 2:**
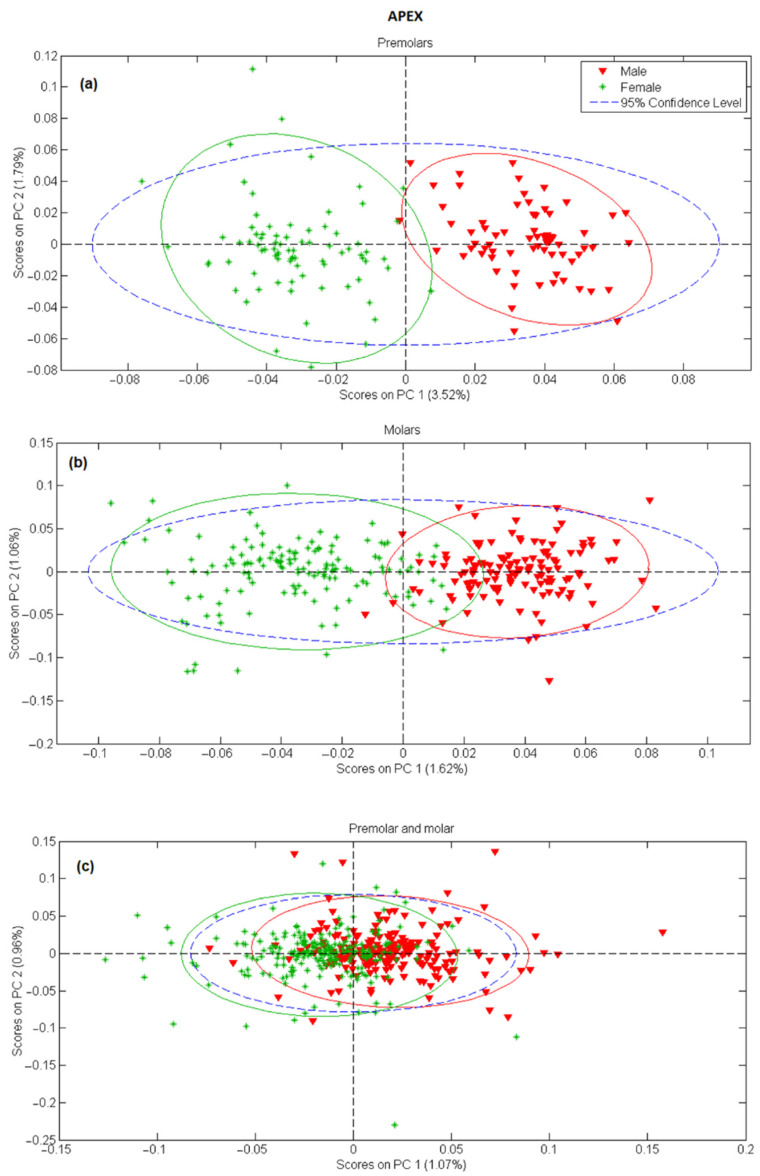
PC1 versus PC2 score plot resulting from the decomposition of Raman data obtained on tooth apex in the 3500–200 cm^−1^ range for: (**a**) premolars, (**b**) molars, (**c**) premolars and molars.

**Figure 3:**
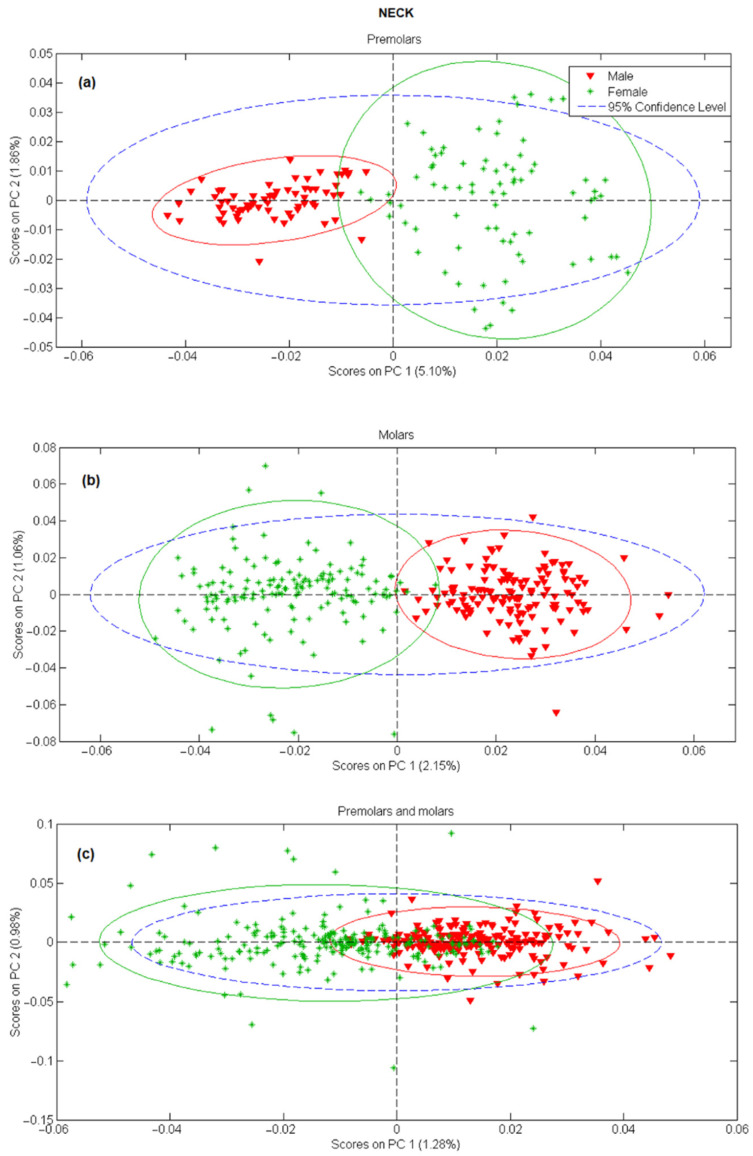
PC1 versus PC2 score plot resulting from the decomposition of Raman data obtained on tooth anatomical neck in the 3500–200 cm^−1^ range for: (**a**) premolars, (**b**) molars, (**c**) premolars and molars.

In the next step, principal component analysis was applied to examine the possibility of separating male and female teeth spectra on a PCA score-score graph. The results for tooth apex and anatomical neck are presented in Figure 2 and Figure 3, respectively, and the PCA scores were calculated separately for tooth spectra of molars and premolars. There is a strong separation between male and female spectra in Figure 2a,b and Figure 3a,b, mostly due to the PC 1 scores. On the other side, when spectra of molars and premolars are combined (Figure 2c and Figure 3c), the ability of PCA to distinguish between female and male spectra is significantly reduced, especially for spectra recorded on the apex. The presented PCA results indicate that detecting gender using the Raman spectroscopy is possible only with the comparison of spectra recorded at the same site on the tooth (apex, anatomical neck) and using the same type of tooth (molar, premolar).

Following these conclusions, eight classification models were built via two different algorithms, support vector machine (SVM) and artificial neural network (ANN), by using calculated PC scores for two tooth types, molars and premolars, and two tooth sites, apex and anatomical neck. First, data were divided into calibration and validation datasets for each combination of tooth type and site, consisting of approximately 83% and 17% of the total number of spectra, respectively. Additionally, as multiple spectra were recorded from every tooth, data partitioning was done by placing all spectra belonging to one particular tooth into the same dataset, either calibration or validation. Second, for both SVM and ANN algorithms, calibration models were calculated using a different number of PCs from 1 to 20, for both recording sites. Third, all calibration models were cross-validated with 10-fold split validation where 70% of the calibration dataset was used for the model and 30% for validation. Finally, the classification success rate, defined as the percentage of correctly classified spectra, was obtained for the validation dataset.

**Figure 4:**
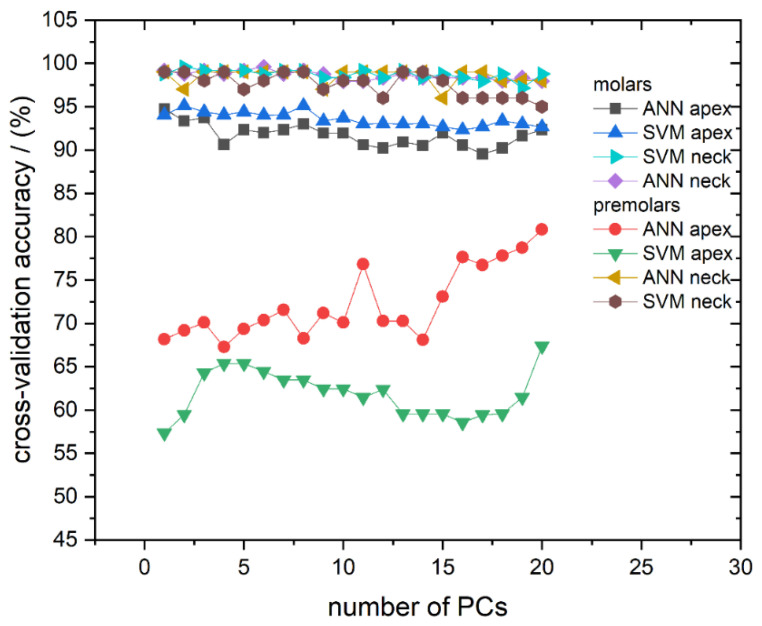
Cross-validation classification accuracy obtained by ANN and SVM in dependence on the number of used PCs for all calculated models: Molar apex and molar anatomical neck; premolar apex and premolar anatomical neck. Lines between symbols were added as a visual aid.

We first address the cross-validation classification results. The classification success rate in the models with a different number of used PCs, for all calculated models, is presented in Figure 4. The cross-validation confusion matrix of the most successful classification models, for spectra recorded on molar and premolar apex obtained by SVM and ANN, respectively, is presented in Table 2. As can be seen in Figure 4, it is evident that most of the computed models have validation accuracy higher than 90%. The exceptions are the models calculated from spectra recorded from apex on premolars, which predominantly have accuracy well below 75%.

**Figure 5:**
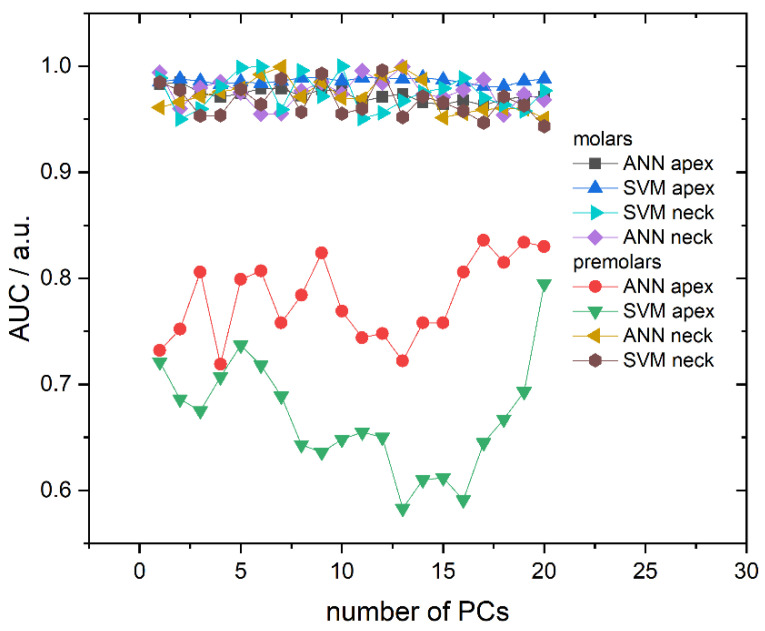
Area Under the ROC Curve (AUC) in dependence on the number of used PCs for all calculated models. Lines between symbols were added as a visual aid.

Area Under the ROC Curve (AUC), dependent on the number of PCs used, is presented in Figure 5 and correlates well with the obtained cross-validation success rate. Namely, AUC values are very high and placed in the 0.965–0.99 interval for all molar models (both algorithms and recording sites) and premolar anatomical neck models, while premolar apex models have much lower AUC values. Additionally, AUC values do not fluctuate significantly with the number of principal components, except in the case of premolar apex models. Finally, calculated diagnostic odds ratio (DOR) and AUC values with their respective 95% confidence intervals presented, in Table 2, point to high diagnostic accuracy in the case of apex molars for both ANN and SVM algorithms.

**Figure 6:**
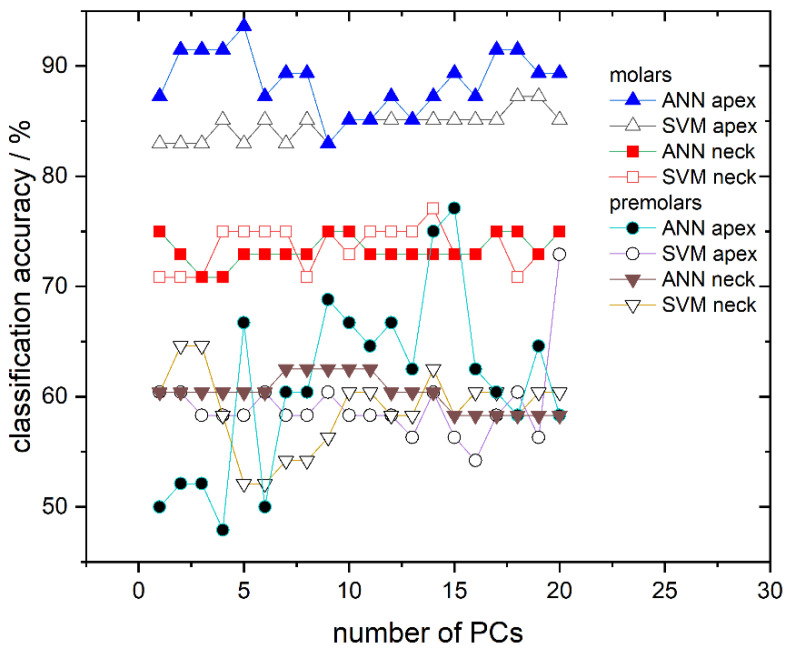
Classification accuracy obtained by ANN and SVM in dependence on the number of used PCs for all calculated models: Molar apex and molar anatomical neck; premolar apex and premolar anatomical neck. Lines between symbols were added as a visual aid.

Finally, the ability of the obtained SVM and ANN models to determine gender utilizing the validation dataset, consisting of 17% unused and “unknown” spectra (not used for the model training), is presented. Results of the SVM and ANN classification are presented in Figure 6 where the success rate dependent on the number of PCs used at different recording sites is shown. The classification success rate for models trained with ANN is represented using full symbols while those trained with SVM use empty symbols. Classification accuracies for the apex on molars are represented with triangles, for the anatomical neck on molars with squares, for the apex on premolars with circles, and for the anatomical neck on premolars with inverted triangles. Based on classification results, molars appear to have much greater potential in gender determination than premolars; the classification success rate between the two tooth types greatly differ (≈70–90% for molars compared to ≈50–70% for premolars). Moreover, it is clearly visible in Figure 6 that, from all tested models, the highest success rate is obtained for molar apex models trained with ANN. Their success rate is dependent on the number of PCs used and several of these models have accuracy higher than 90%. In addition, models trained with the same spectra but with the SVM algorithm have slightly lower accuracy, which falls in the range of 82–87% depending on the number of used PCs. On the other hand, molar anatomical neck models exhibit lower classification potential with a success rate of 70–77%, being almost independent of the used algorithm. Finally, premolar apex and premolar anatomical neck models have success rates grouped around 60%.

**Figure 7:**
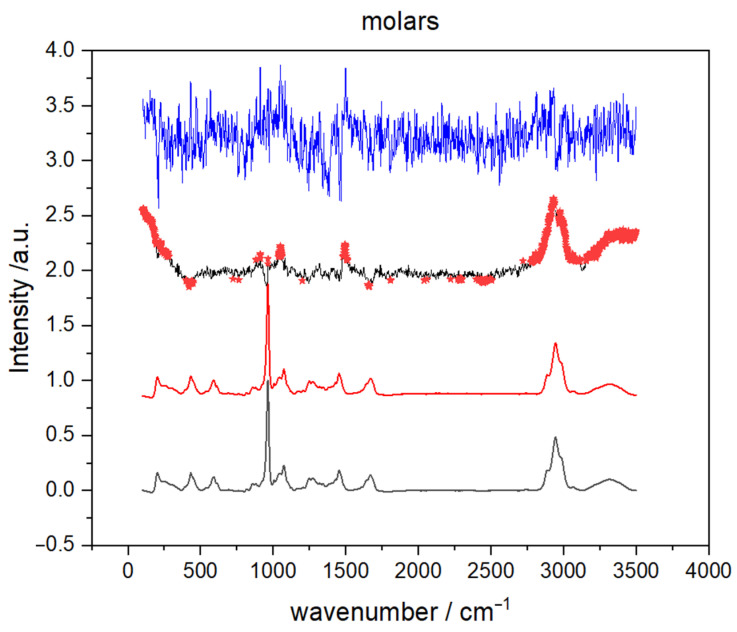
The average Raman spectra of molar apex male (grey line) and female (red line) teeth obtained in the spectral range between 3500–200 cm^−1^. The differential spectrum of average male and female spectra (male-female, black line) enhanced with student *t*-test result (red stars) and corresponding PC1 loadings (blue line).

To identify the parts of Raman spectra that contributed the most to the separation between male and female teeth, averaged spectra of teeth with spectral difference enhanced with Student’s *t*-test (STT) result and PC loadings were compared. The analysis was restricted to molar apex spectra because, according to hitherto presented findings, premolars exhibit much lower gender classification potential. The results are shown in Figure 7 where two spectra (black and red) on the bottom represent average spectra of male and female molar apex, respectively. Moreover, the differential spectrum (male–female), in which red stars mark specific parts of the spectrum where the Student’s *t*-test (*p* < 0.01) showed a statistically significant difference between average male and female spectrum, is presented just below the PC 1 loadings (molar apex), found at the top of Figure 7. The first principal component was solely used because it exhibited the strongest difference between two gender groups in the analysis of PCA scores. By comparing PC 1 loadings with differential STT spectrum it is clear that the wavenumbers at which STT showed statistical significance coincide with those where loadings have more pronounced values. Statistically significant differences were discovered for the bands at 953 cm^−1^ (proline), 1070 cm^−1^ (CO_3_ stretching), 1450 cm^−1^ (CH_2_ bending), 1665 cm^−1^ (Amide I), and in the range of CH_2_ stretching vibrations (between 2880 and 2980 cm^−1^).

Statistical significance of these vibrational bands indicates a certain alteration in the organic matrix so we calculated and compared intensity ratios (PO_4_ (960 cm^−1^)/amid I (1665 cm^−1^) and PO_4_ (960 cm^−1^)/nCH_2_ (2940 cm^−1^)), which are the standard used for determination of minerals to matrix ratio [32,33]. For each recorded Raman spectrum, we read off intensities, calculated the aforementioned ratios, and then used a Student’s *t*-test test. For spectra recorded on molar apex, STT showed that the difference between average values for male and female teeth is statistically significant (*p* < 0.05). Thus, results in Table 3 are an indication that the difference in male and female spectra, for spectra recorded on the molar apex, might be connected with the changes in the mineral-to-matrix ratio.

## 4. Discussion

Our previous research on age determination using Raman tooth spectra [21] has shown that the ability to correctly determine age is lower for female compared to male teeth. This result provided the idea for the present study in which the multivariate models for gender recognition based on Raman spectra were built and tested. In this work, we focused solely on molars and premolars because these teeth are generally extracted more often making it much easier to obtain a sufficient number of samples for analysis. The Raman spectra were recorded on two distinct spots on the tooth, the apex and the anatomical neck. We also recorded Raman spectra on enamel but these findings were not included in this study; the enamel is predominantly inorganic, resulting in a relatively poor spectrum [26] whose properties are also strongly influenced by one’s lifestyle habits [21]. Consequently, enamel spectra are less suitable for classification modeling and are thus not a part of this study.

In order to access the potential of Raman spectroscopy in gender recognition, decomposition and reduction of spectroscopy data were first carried out by means of PCA [34]. In Figure 2 and Figure 3, general clustering of data was evident, providing a strong indication that PC scores could successfully be used in determining gender, especially in the case when different tooth types (molars and premolars) are modeled separately. On the other hand, clustering of the spectra is less pronounced and significant overlap around the origin is evident when PC models include both molars and premolars. It can be reasoned that PC1 scores account for gender-related but also some other, gender nonrelated, differences in the Raman spectra. Thus, in the case of different tooth types, spectral variances induced by slight changes in chemical composition or structure could suppress gender-related effects resulting in less successful clustering. An excitation laser penetrates deep inside the teeth meaning that the resulting Raman spectra are a mixture of vibrational bands present in both dentin and cementum. If the cementum layer is thicker, more information will be collected from cementum compared to dentin. Cementum layer in the premolars is generally thinner than in molars [35] thus we can expect that more information about cementum is present in Raman spectra of molars. If we assume that gender information is stored in cementum, spectra recorded on premolars will have that information screened with dentin vibrational bands. This mixture of vibrational bands would definitely modify structural and chemical information contained in Raman spectra making the classification process much more uncertain.

Better classification results can be achieved when applying classification algorithms. Therefore, apart from revealing spectra clustering, PCA was also used for data reduction since Raman spectra possess too much data for practical classification. Hence, the first 20 PCs were used to calculate classification models. To reduce the possibility of overfitting but also to test the classification accuracy of different classification algorithms, both SVM and ANN were utilized. Additionally, as briefly mentioned in Materials and Methods, our models for gender determination did not include the data about pathological processes in order to simulate a realistic forensic scenario in which these variables are unknown. Cross-validation accuracy and AUC showed that both algorithms give similar results for both observed spots (apex and anatomical neck) on two tooth types; however, some differences were observed between molars and premolars. Examples of cross-validation for models generated using Raman spectra recorded on the apex are shown in Table 2 with confusion matrices. It is conspicuous from Figure 4 and Figure 5 that cross-validation accuracy and AUC values for apex on premolars are much smaller compared to rest of the results. The decrease of classification accuracy for premolar apex could be connected with differences in structural and/or chemical composition of these teeth or by the simple fact that the number of molars used in the experiment is much bigger than the number of premolars, as typically, a higher number of spectra in the model makes the model more reliable. Due to the relatively small number of premolar samples, the influence of possible outliers (the spectra of a few problematic teeth, which have been exposed to some unusual life impacts) on the calculated models might be rather strong. If the limited number of premolar samples was the problem, it can be easily overcome by their increase in future investigations.

It is also important to note the high consistency of the results obtained using the models calculated with both applied algorithms. This consistency excludes the possibility of overfitting and confirms the quality of the used methodology. The dependence of AUC on tooth type is also in accordance with the obtained results for classification accuracy. Namely, higher AUC values are obtained for molars compared to premolars and ANN again proves to be superior to SVM. AUC values for molars are close to 1 and 0.9 for ANN and SVM, respectively, while for premolars, AUC values are lower for both ANN and SVM.

Because the number of molar specimens was greater than that of the premolars, the classification accuracy of models with an unknown dataset was done only for molars, but for both recording sites, apex and anatomical neck (Figure 6). The best results were obtained using ANN and apex spectra where the percentage of accurately recognized spectra was greater than 90%. Moreover, it is shown that the number of used PCs affects the performance of the model. We attribute this behavior to different contributions of individual components and also believe that optimizing the selection of PCs used for modeling could further increase the success of gender recognition. Since Rapidminer allows the optimization of features, which contribute most to the model, i.e., optimization of principal components, we believe that this approach could enhance the classification accuracy of our models. However, such optimization of parameters requires a lot of computing time and power, so in this work, where only the possibility of gender recognition using Raman spectra is presented, it was not applied. Models built using the SVM algorithm and apex gave just slightly worse gender recognition accuracy compare to ANN, which further confirmed the possibility of gender recognition by the Raman spectra of the teeth.

Finally, let us address the issue of different classification accuracy of the two distinct tooth sites, the apex and anatomical neck. The classification accuracy for the molar anatomical neck is lower than the molar apex, from 70 to 75%, and approximately the same for both SVM and ANN algorithms. Apex and anatomical neck are covered with different types of cementum, the apex with cellular and the anatomical neck with a thinner layer of acellular cementum [36]. It is possible that gender recognition information is stored within cellular cementum. That assumption is suggested from the analysis of recorded spectra, Figure 7, where the difference in male and female spectra was related to the difference in the mineral-to-matrix ratio (Table 3). Organic material is more present in cellular than in acellular cementum [36] and its structure and composition are under the influence of gender-dependent life events [37]. There are no literature data on the differences between male and female cementum on the molecular level but it is reasonable to assume that composition of the organic matrix of cementum is slightly different in male and female teeth and thus consistent with changes observed in the presented results (Table 3). Moreover, the cementum covering the anatomical neck is exposed to various environmental factors that affect its composition, which in turn reflects on Raman spectra. Over a lifetime, the gingival attachment is often retracted from the anatomical neck, exposing it to environmental conditions of oral cavity, including the effects of oral bacteria and mechanical abrasion. This often leads to the damage of the thin cementum layer at the anatomical tooth neck and the consequent exposure of the underlying dentin. The exposed dentin shows a defensive response by hypermineralization, tubular occlusion, and a general increase in mineral content. All of these processes can reflect on Raman spectra collected at the anatomical neck, increasing their inter-individual variability [21].

The differences between male and female cementum could also originate from variations in masticatory loading. Namely, higher masticatory loading can cause higher dental cementum deposition [35] and could thus explain the differences between male and female teeth. Furthermore, the difference in classification potential of molars and premolars could also be rationalized with this assumption; molars are exposed to higher masticatory loading causing higher dental cementum deposition and thus exhibiting higher classification potential. However, this is only an assumption, as although cementum deposition can be subjected to masticatory loading, which is generally higher in males [38], there is no convincing evidence for a measurable gender-specific response of cementum-producing cells to these forces [39].

## 5. Conclusions

In this work Raman spectra of teeth were recorded in order to extract information about the owner’s gender. Raman spectra were analyzed with PCA, and classification models were built with classification algorithms SVM and ANN. It was observed that the accuracy of classification models depends both on the tooth type (molar and premolar) and recording site (anatomical neck and apex) on the tooth. The best classification accuracy (>90%) was achieved with spectra recorded from (apex on molars) while classification models built with spectra recorded from premolars and from anatomical neck had lower classification accuracies. The comparison of two used classification algorithms showed that models built with ANN result in slightly better classification accuracies especially for models built with spectra recorded on molar apex. Moreover, classification accuracy varies with the number of used PCs suggesting that optimizing the selection of used principal components might improve the final classification accuracy of the model.

## Figures and Tables

**Table 1:** Assignment of major bands found in Raman spectra of teeth [27,30,31].

Assignment	Wavenumber [cm^−1^]
ν(CH_2_) symmetric stretching	2979
ν(CH_2_) symmetric stretching	2940
ν(CH_2_) asymmetric stretching	2883
ν(C=O) Amide I	~1665
δ(CH_2_) bending mode δ(N-H) Amide III	~1450
	~1242
ν_1_ (CO_3_ type B) symmetric stretching	1069
ν_1_ (PO_4_) symmetric stretching	960
ν(CC); ν_2_(CO_3_) hydroxyproline	876
δ (CCH) aromatic; ν_2_(CC) proline	~853
ν_4_ (PO_4_) asymmetric bending	590
ν_2_ (PO_4_) symmetric bending	~431

**Table 2:** Cross-validation confusion matrices for models with highest obtained success rate (apex models). AUC values with their respective 95% confidence intervals and calculated DOR values are also presented.

Molars						Premolars					
**ANN**			**SVM**			**ANN**			**SVM**		
1 PC; Accuracy 94.77%			8 PCs; Accuracy 95.12%			20 PCs; Accuracy 80.89%			20 PCs; Accuracy 67.52%		
AUC = 0.98 ± 0.02 DOR = 329			AUC = 0.99 ± 0.01 DOR = 413			AUC = 0.83 ± 0.07 DOR = 18			AUC = 0.80 ± 0.07 DOR = 4.39		
**Confusion Matrix:**			**Confusion Matrix:**			**Confusion Matrix:**			**Confusion Matrix:**		
True	Male	Female	True	Male	Female	True	Male	Female	True	Male	Female
Male	130	7	Male	133	9	Male	60	17	Male	51	29
Female	8	142	Female	5	140	Female	13	67	Female	22	55

**Table 3:** Determination of the mineral-to-matrix ratio, as indicated by Raman intensity ratio of the corresponding bands, for spectra recorded on molar apex.

PO_4_ (960 cm^−1^)/Amide I (1665 cm^−1^)		PO_4_ (960 cm^−1^)/CH_2_ (2940 cm^−1^)	
Male	Female	Male	Female
7.255	7.436	2.23	2.15

## Data Availability

Not applicable.

## Appendix A

**Table A1:** Characteristics of teeth used in this study.

Specimen No.	Gender	Tooth Type According to ISO 3950 Notation	Donor Age (Years)	Time between Extraction and Spectra Collection (Years)	Reason for Extraction
1	M	14	15	0.2	ortho
2	M	14	16	3.5	ortho
3	M	35	16	3.5	endo
4	M	47	22	0.2	endo
5	M	18	23	0.1	imp/ret
6	M	37	31	0.1	endo
7	M	37	32	0.2	endo
8	M	47	35	0.3	endo
9	M	27	44	0.1	endo
10	M	45	45	2.9	endo
11	M	14	47	4.2	endo
12	M	48	49	0.3	perio
13	M	25	52	3.9	perio
14	M	46	52	0.2	endo
15	M	47	54	0.3	perio
16	M	15	55	3.7	perio
17	M	27	55	4	perio
18	M	37	58	4.2	perio
19	M	17	58	4.2	endo
20	M	17	64	0.4	perio
21	M	44	64	0.1	perio
22	M	36	65	0.3	endo
23	M	17	67	0.2	endo
24	M	27	67	0.1	perio
25	M	45	68	4	perio
26	M	36	76	0.3	endo
27	F	24	11	4	ortho
28	F	36	17	0.5	endo
29	F	35	23	0.3	ortho
30	F	36	23	0.4	endo
31	F	47	23	0.5	endo
32	F	18	26	5.5	endo
33	F	28	29	0.4	imp/ret
34	F	28	31	0.3	endo
35	F	24	35	4.4	endo
36	F	28	35	0.4	endo
37	F	27	38	0.6	perio
38	F	18	39	0.6	endo
39	F	45	39	3.8	perio
40	F	48	39	0.3	imp/ret
41	F	37	46	0.6	endo
42	F	36	48	0.1	endo
43	F	35	49	3.4	perio
44	F	44	49	3.7	perio
45	F	27	51	0.5	perio
46	F	25	53	3.9	perio
47	F	37	55	0.5	endo
48	F	27	56	0.5	perio
49	F	34	58	0.2	perio
50	F	37	58	0.6	perio
51	F	45	62	4	perio
52	F	17	64	0.5	endo
53	F	45	65	4.1	perio
54	F	26	66	0.6	perio
55	F	37	73	0.6	endo

Reasons for extraction: Ortho = orthodontic extraction, endo = failed endodontic treatment or inability to perform endodontic treatment, perio = extraction due to advanced periodontitis, imp/ret = impacted or retained tooth.
